# Post‐Match Recovery Responses in Italian Serie A Youth Soccer Players: Effects of Manipulating Training Load 48 h After Match Play

**DOI:** 10.1002/ejsc.12297

**Published:** 2025-04-20

**Authors:** Alberto Franceschi, Mark A. Robinson, Daniel J. Owens, Thomas Brownlee, Darragh R. Connolly, Aaron J. Coutts, Duccio Ferrari Bravo, Kevin Enright

**Affiliations:** ^1^ Research Institute for Sport and Exercise Sciences Liverpool John Moores University Liverpool UK; ^2^ Sport Science and R&D Department Juventus Football Club Torino Italy; ^3^ School of Sport, Exercise and Rehabilitation Sciences University of Birmingham Birmingham UK; ^4^ Faculty of Health School of Sport, Exercise and Rehabilitation Human Performance Research Centre University of Technology Sydney Sydney Australia

**Keywords:** fatigue, football, performance, recovery, training load

## Abstract

This study described the recovery responses following match play and examined the effects of manipulating training load 48 h post‐match in Italian Serie A youth soccer players. Forty‐eight players were assessed using the countermovement jump (CMJ), isometric posterior‐chain muscle test (IPC), muscle soreness and fatigue before (pre) and after (0.5 h post, 48 h post and 72 h post) a match. At 48 h post‐match, players were randomly assigned to a complete training (CT; *n* = 26) or a reduced training (RT; *n* = 22) group. Recovery differences were analysed between time points and training groups, with training loads quantified on match day (MD) and match day plus two (MD + 2). Recovery measures were impaired immediately post‐match (*p* < 0.05). IPC and muscle soreness demonstrated incomplete recovery 48 h post‐match (*p* < 0.05), whereas CMJ and fatigue returned to baseline (*p* > 0.05). Training load on MD did not differ between groups (*p* > 0.05), whereas the CT group had higher load on MD + 2 compared to RT (*p* < 0.05). A significant time × group interaction was observed for CMJ height and IPC measures (*p* < 0.05), with reductions in physical performance observed in the CT group from 48 to 72 h post‐match (*p* < 0.05). A youth soccer match acutely impaired physical performance and recovery status, with prolonged and incomplete recovery of hamstring force and elevated muscle soreness 48 h post‐match. A high‐volume and high‐intensity session administered 48 h post‐match negatively influenced physical performance compared to a moderate training session.

**Summary**

- Italian Serie A youth soccer players exhibited significant acute reductions in physical performance and perceptual recovery after match play. A prolonged and incomplete recuperation was evident for hamstring muscle strength and the perception of muscle soreness 48 h post‐match.
- Engaging in high‐volume and high‐intensity training sessions 2 days after a youth soccer match may negatively affect the recovery process and diminish performance capabilities. Adopting lighter training sessions during this time frame could alleviate adverse recovery effects and support performance enhancement.
- Monitoring recovery post‐match, including assessments of hamstring muscle strength and muscle soreness, alongside both external and internal training load measures, can assist sports scientists and coaches in identifying recovery requirements and optimising training programmes for youth soccer players.

## 1 Introduction

Effective management of fatigue and recovery following training sessions and competitions is a primary responsibility of sports scientists and coaches (Skorski et al. 2019; Thorpe et al. 2017). Recovery is a multifaceted and complex process that involves various restorative measures over time, serving as a vital part of the overall training program (Kellmann et al. 2018; Mujika et al. 2018). The training process consists of the systematic execution of physical activities, encompassing both external and internal training loads, along with their respective training responses, which can be either beneficial or detrimental, and can lead to acute or chronic effects that influence sports performance (Jeffries et al. 2022). To maximise positive training impacts while minimising negative interactions with training adaptations, it is advised to adopt a structured, periodised, specific and individualised strategy for training and recovery monitoring (Coutts et al. 2018; Impellizzeri et al. 2019; Wiewelhove et al. 2024).

The physical demands of soccer matches cause fatigue, leading to changes in physiological and biochemical balance, reduced neuromechanical function and alterations in psychological perceptions (Brownstein et al. 2017; Krustrup et al. 2006; Mohr et al. 2023; Rampinini et al. 2011). Fatigue is characterised by a decrease in performance and an altered perception of effort that maintains an individual's integrity (Enoka and Duchateau 2016). In soccer, diminished hamstring muscle function and countermovement jump (CMJ) performance, along with increased muscle soreness and creatine kinase levels, indicate a prolonged recovery period. Players' physiological states and performance capacities remain significantly impaired 48 h post‐match, with some alterations evident up to 72 h after the match in professional players (Silva et al. 2018). Although numerous studies have characterised recovery responses in adult players, limited information exists on post‐match recovery in youth soccer players.

Significant changes in physical performance (e.g., vertical jumps and hamstring strength), physiological markers (e.g., metabolic, inflammatory and muscle damage) and perceptual measures (e.g., muscle soreness and fatigue) have been documented after youth soccer matches (Barreira et al. 2023; Bromley et al. 2021; Constantine et al. 2019; de Hoyo et al. 2016; De Ste Croix et al. 2019; Fornaziero et al. 2023; Franceschi et al. 2023; Izquierdo et al. 2020; Martin‐Garetxana et al. 2024; Romagnoli et al. 2016; Wollin et al. 2017). Research showed inconsistent results regarding the recovery of physical performance 2 days post‐match in under‐19 players competing in 90 min games (Barreira et al. 2023; de Hoyo et al. 2016; Romagnoli et al. 2016). More recently, Springham et al. (2024) examined the time course of recovery to under‐18 English Premier League soccer matches, reporting match‐induced reductions in isometric strength measures (posterior‐chain, adductor and abductor muscles) which normalised between 2 and 3 days post‐match. Considering that youth players generally engage in shorter matches of 80 or 70 min, depending on their age group (Palucci Vieira et al. 2019), it is conceivable that their recovery responses might vary in magnitude and duration compared to adult players.

Following competitive matches, soccer players often employ several recovery and training methods to reduce fatigue and speed up recuperation (Field et al. 2021). Despite extensive research on recovery strategies in soccer (Altarriba‐Bartes et al. 2020; Calleja‐González et al. 2021; Querido et al. 2022), the success of these methods in improving recovery has been uncertain or minimal, with significant variability in individual responses (Wiewelhove et al. 2024). Thus, it is crucial to focus on key aspects of recovery and training, such as adequate sleep, proper nutrition and management of training load, to optimise recovery and performance capacity (Driller and Leabeater 2023). Practitioners working with team sport players prioritise recovery in the initial 48 h post‐match by limiting intense training sessions within this time frame (Cross et al. 2019). However, previous research conducted with youth soccer players has documented that adolescent players can experience intense training loads 2 days following match play (Franceschi et al. 2024; Wrigley et al. 2012). Therefore, gaining insights into how different training loads impact recovery when players resume training 48 h post‐match would assist practitioners in optimising training programs that balance recovery needs with sport‐specific preparation for youth soccer players.

Manipulation of training load during the competitive microcycle involves adjusting the frequency, duration and intensity of training sessions to achieve desired training effects (Douchet et al. 2024; Slattery et al. 2012; Thorpe et al. 2017). Prior studies indicate that an active recovery session conducted 48 h post‐match more effectively restores knee flexor muscle strength, decreases creatine kinase levels and reduces muscle soreness compared to a typical training session in highly trained youth soccer players (Trecroci et al. 2020, 2021). Nevertheless, information on the impact of various soccer‐specific training interventions given when adolescent players return to training 2 days after a match remains limited. As soccer players can exhibit altered recovery states and higher perceived fatigue 48 h after matches (Di Salvo et al. 2023; Silva et al. 2018), it is important to prescribe an appropriate training stimulus early in the microcycle to facilitate the intended recovery of performance and ensure proper preparation for forthcoming matches.

Given this context, this study aimed to (1) describe the recovery responses up to 48 h post‐match and (2) to examine the effects of training load manipulation 48 h after match on these responses, using two training interventions with distinct volume and intensity in Italian Serie A youth soccer players.

## 2 Methods

### 2.1 Subjects

Forty‐eight youth soccer players belonging to the academy squads of a professional soccer team in the Italian Serie A participated in this study (age: 15.4 ± 0.9 years; height: 178.0 ± 6.2 cm; body mass: 66.5 ± 7.3 kg; percentage adult height: 98.0 ± 1.6% and maturity offset: 1.8 ± 0.8 years). Players were free from injury and illness and participated in an average of 8–10 h of training and a competitive match per week. Players would be classified as highly trained (Tier 3) or elite (Tier 4) (McKay et al. 2022). Parents or legal guardians provided written informed consent prior to the commencement of the study. The study was ethically approved by a University Research Ethics Committee (23/SPS/006) and was conducted in accordance with the Declaration of Helsinki.

### 2.2 Experimental Design

**FIGURE 1:**
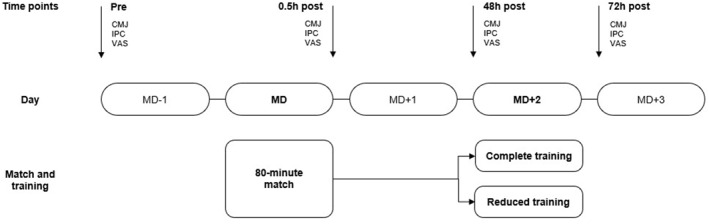
Study design overview. Physical performance (CMJ and IPC) and perceptual recovery measures (VAS for muscle soreness and fatigue) were collected during the microcycle at four time points before (pre) and after (0.5 h post, 48 h post and 72 h post) a match; 2 days after match, players were assigned to either a complete training group (CT: 100 min session and *n* = 26) or a reduced training group (RT: 70 min session and *n* = 22). + and − represent the training days with respect to the distance to the match; CMJ: countermovement jump; h: hours; IPC: isometric posterior chain; MD: match day; VAS: visual analogue scale.

Players were assessed at four time points during a microcycle: −24 h before the match (pre), immediately after the match (0.5 h post) and 2 and 3 days after the match (48 h post and 72 h post). At each time point, physical performance (countermovement jump and isometric posterior‐chain muscle test) and perceptual recovery measures (visual analogue scales for muscle soreness and perceived fatigue) were collected to evaluate recovery responses. Baseline testing sessions were preceded by 48 h of rest. Three friendly matches were organised and each participant played an 80 min friendly match play (MD; 2 × 40 min, 105 × 68 m, artificial turf). The matches were preceded by a 20 min standardised warm‐up. Players had to complete the entire match to be included in the analyses. On the day following the match (MD + 1), players had a rest day, without exposure to any physical activity. Two days after the match (MD + 2), players were assigned to either a complete training group (CT: 100 min session and *n* = 26) or a reduced training group (RT: 70 min session and *n* = 22) using a randomised parallel group design. Training and match loads were quantified using global navigation satellite systems (GNSS), heart rate (HR) sensors and session rating of perceived exertion (sRPE). Players were familiarised with all testing and monitoring procedures and were instructed to maintain their normal dietary intake. A schematic overview of the study design is depicted in Figure 1.

### 2.3 Procedures

*Quantification of training and match loads*: External load was quantified using GNSS (Apex Pro Series, 10 Hz, STATSports, Newry, Northern Ireland). This tracking system provides valid and reliable estimates of distance and velocity during team sports activities, including linear and multidirectional movements (Beato et al. 2023; Coutts and Duffield 2010). At the end of each session, data were downloaded and then cropped from the start of the first activity to the end of the last drill or the end of match play on the Sonra software (version 4.1.31, STATSports, Newry, Northern Ireland). The average number of satellite signals was 18 ± 4 and the horizontal dilution of precision was 0.5 ± 0.1. Internal load was quantified using HR sensors (Polar H10, Polar, Kempele, Finland) and sRPE. HR traces of each player were visually examined on the Sonra software to verify recording quality. In case of signal loss or multiple drops within an experimental session, HR data were removed (12% of the initial dataset). sRPE, measured using Borg's CR‐10 scale (Foster et al. 2001; Impellizzeri et al. 2004), was collected around 30 min following each training session and match. sRPE training load (sRPE‐TL) for each session was subsequently calculated by multiplying the player's sRPE by the duration of the session (Foster et al. 2001). In line with previous research (Franceschi et al. 2024; Hannon et al. 2021; Wrigley et al. 2012), the following training and match load measures were selected for the analyses: total distance (m), distance covered > 15 km/h (m), distance covered > 20 km/h (m), distance covered > 25 km/h (m), maximal speed (km/h), accelerations > 3 m/s^2^ (count) and decelerations < −3 m/s^2^ (count), average heart rate (bpm), peak heart rate (bpm), time spent > 85% HR_max_ (min), time spent > 90% HR_max_ (min), sRPE (arbitrary units, AU) and sRPE training load (AU).

*Training load interventions*: Players were randomly assigned to either a complete training (CT: 100 min) or a reduced training (RT: 70 min) session. Session duration was calculated from the start of the first activity to the end of the last drill. The definition of complete training reflects the typical training load sustained by Italian youth soccer players 2 days after match play as previously reported by our group (Franceschi et al. 2024). The 30 min between‐group difference was selected to align with practical aspects of training load management adopted in soccer. The CT session consisted of warm‐up (15 min), technical passing drills (15 min), ball possession games (15 min; 6 vs. 6 with floaters; 40 × 40 m), small‐sided games (25 min; 5 vs. 5 with goalkeepers and regular target; 35 × 40 m), position‐specific technical drills (15 min) and high‐intensity intermittent runs (5 min; 15 s work and 15 s rest). The RT session consisted of warm‐up (15 min), technical passing drills (15 min), ball possession games (15 min; 6 vs. 6 with floaters; 40 × 40 m) and position‐specific technical drills (15 min). The RT session was planned with a shorter duration, volume and intensity by including technical‐tactical drills with limited impact and physical demands. Experimental training sessions were also monitored with live tracking technology using Sonra Live App (version 2.8, STATSports, Newry, Northern Ireland). This tracking system has been shown to provide valid data both in real‐time and post‐activity in team sports (Johnston et al. 2020).

*Countermovement jump (CMJ) test:* Players performed three CMJ trials with ∼20 s rest between trials on a force platform (ForceDecks Dual Force Plate System FD lite, VALD Performance, Newstead, Australia), recording with a sampling rate of 1000 Hz and using methods previously described (Barillas et al. 2021; McMahon et al. 2018). Before the commencement of each jump, players were advised to stand upright, with their hands on their hips and their feet hip‐shoulder width apart. Once the starting position was adopted, players remained as still as possible for at least 3 s before the start of the trial for the collection of the player's body weight. During the countermovement, players were instructed to rapidly squat to their preferred depth and immediately jump as high as possible and as quick as possible, with no knee or hip flexion during the flight phase, maintaining the hands on the hips. Finally, players were encouraged to ‘absorb’ the landing by flexing at the hips, knees and ankles after impacting the force platform. Jump height (cm), peak power (W), force at zero velocity (N) and reactive strength index (RSI) modified (m/s) were analysed. These measures were chosen due to their suitability in the context of performance profiling and fatigue monitoring in soccer (Bishop et al. 2023; Franceschi et al. 2023).

*Isometric posterior chain (IPC) lower‐limb muscle test:* Players performed a 3 s maximal contraction with ∼30 s of rest between trials into a force platform (ForceDecks Dual Force Plate System FD lite, VALD Performance, Newstead, Australia), recording with a sampling rate of 1000 Hz similar to methods previously reported (McCall et al. 2015). Before each testing trial, players laid in a supine position with their knee at 90° of flexion, with their calcaneus on the centre of the force platform and the nontest leg extended alongside a box at an appropriate height for each participant (i.e., lower shank to be parallel to the floor). Players were instructed to push their heel maximally down into the force platform. The tester ensured a correct position of both legs and pressure was applied to the contralateral hip to control participant posture (i.e., keeping the buttocks, hips and head on the floor). Players were required to repeat trials if a measurement error in posture was observed. Three trials on each limb were executed with ∼30 s rest between trials. Peak force was quantified for each trial. Moment arm length was measured from the joint axis of rotation to the point of application of the force and peak torque was calculated by multiplying the peak force by the length of the moment arm. Peak force dominant leg (N), peak force nondominant leg (N), peak torque dominant leg (Nm) and peak torque nondominant leg (Nm) were analysed.

*Perceptual scales*: At each measurement time point, players rated their perceived level of muscle soreness and fatigue using visual analogue scales (VAS; 0 = no soreness/fatigue; 10 = extreme muscle soreness/fatigue; Abbott and Clifford 2022; Cross et al. 2023). Both measurements were reported in centimetres.

### 2.4 Statistical Analysis

Data are presented as mean ± standard deviation (SD) or as mean ± 95% confidence intervals (CIs). Analysis of training load and recovery response measures commenced following the assessment of normality using the Shapiro–Wilk test and of equality of variance using Levene's test. Independent *t*‐tests were performed to analyse the differences in external and internal load between the training groups on MD (match) and MD + 2 (training load interventions). To analyse post‐match recovery responses, a one‐way repeated measures analysis of variance (ANOVA) was performed for each recovery measure to examine differences between time points (pre, 0.5 h post and 48 h post). Sphericity was assessed using the Mauchly test and if violated, the Greenhouse–Geisser correction was used to adjust the degrees of freedom. Where a significant main effect was present, a Bonferroni post hoc analysis was conducted to locate specific differences. A separate parallel analysis of variance, a 2 × 2 mixed ANOVA was performed to investigate the effects of the two training load interventions on recovery responses between time points (48 h post and 72 h post) and training groups (complete training and reduced training). Within this analysis, these time points reflect pre (48 h post) and post (72 h post) training load intervention measurements. The magnitude of differences in training load and recovery measures was also assessed using Cohen's *d* effect size (*d*) and was interpreted as follows: trivial ( < 0.20), small (0.20–0.59), moderate (0.6–1.19), large (1.20–1.99) and very large ( > 2.0) (Hopkins et al. 2009). Statistical significance was set with alpha < 0.05. Statistical analyses were performed using the JASP statistical software (JASP 0.18.3 version, University of Amsterdam, Amsterdam, The Netherlands).

## 3 Results

### 3.1 Match and Training Load Characteristics

No between‐groups differences were observed on MD, with groups exposed to similar external and internal load (*p* > 0.05; *d*: ranged from trivial to small; Table S1). On MD + 2, the complete training group had significantly higher training loads than the reduced training group for all external and internal load measures (*p* < 0.05, *d*: ranged from moderate to very large; Table 1).

**TABLE 1:** Training load interventions characteristics on MD + 2: descriptive, inferential and effect size statistics of external and internal load measures between complete and reduced training groups.

	Complete training (*n* = 26)	Reduced training (*n* = 22)	Mean difference (95% CI)	*p*‐value	Cohen's *d* (95% CI)	Interpretation
External load						
Total distance (m)	7842 ± 839	4371 ± 816	3471 (2988; 3955)	< 0.001	4.19 (3.16; 5.21)	Very large
Distance > 15 km/h (m)	1178 ± 374	330 ± 168	848 (674; 1022)	< 0.001	2.85 (2.03; 3.65)	Very large
Distance > 20 km/h (m)	180 ± 131	58 ± 44	122 (63; 180)	< 0.001	1.21 (0.58; 1.82)	Large
Distance > 25 km/h (m)	12 ± 10	3 ± 5	9 (4; 13)	< 0.001	1.06 (0.45; 1.66)	Moderate
Maximal speed (km/h)	26.9 ± 2	24.8 ± 2.1	2.1 (0.9; 3.3)	< 0.001	1.02 (0.41; 1.62)	Moderate
Accelerations > 3 m/s^2^ (count)	70 ± 17	39 ± 11	31 (23; 40)	< 0.001	2.16 (1.44; 2.87)	Very large
Decelerations < −3 m/s^2^ (count)	68 ± 16	33 ± 14	35 (27; 44)	< 0.001	2.37 (1.62; 3.11)	Very large
Internal load						
Average HR (bpm)	151 ± 12	140 ± 19	10 (−0; 20)	0.054	0.65 (−0.01; 1.30)	Moderate
Peak HR (bpm)	196 ± 7	188 ± 15	8 (1; 16)	0.024	0.77 (0.10; 1.43)	Moderate
Time > 85% HR_max_ (min)	29 ± 12	12 ± 11	16 (9; 24)	< 0.001	1.36 (0.67; 2.03)	Large
Time > 90% HR_max_ (min)	12 ± 9	6 ± 7	6 (1; 12)	0.014	0.80 (0.16; 1.42)	Moderate
sRPE (AU)	4.1 ± 0.8	3.3 ± 0.8	0.8 (0.3; 1.219)	0.002	0.96 (0.36; 1.56)	Moderate
sRPE‐TL (AU)	348 ± 86	195 ± 103	153 (99; 209)	< 0.001	1.63 (0.97; 2.28)	Large

*Note:* Data are mean ± SD.

Abbreviations: AU, arbitrary units; bpm, beats per minute; CI, confidence interval; HR, heart rate; m, metres; *n*, number; TL, training load.

### 3.2 Recovery Responses Following Match Play

**FIGURE 2:**
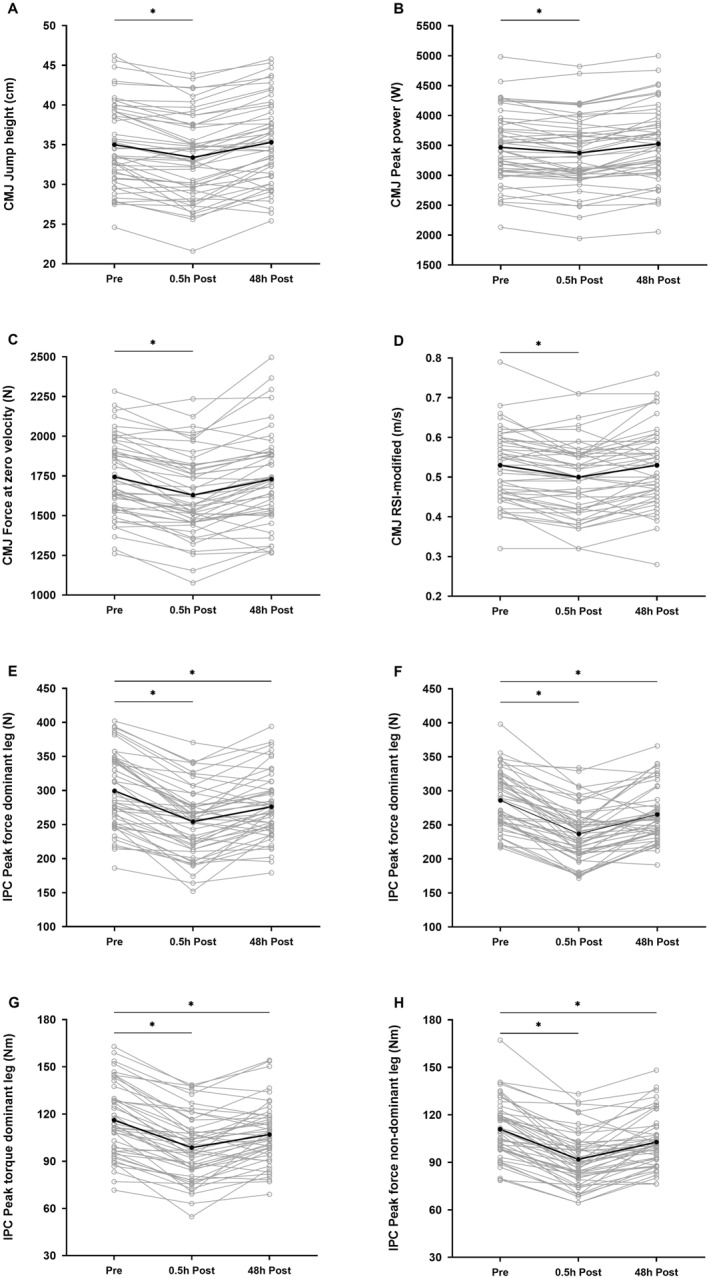
Individual responses of countermovement jump (A) jump height, (B) peak power, (C) force at zero velocity, (D) RSI‐modified and isometric posterior chain (E) peak force dominant leg, (F) peak force nondominant leg, (G) peak torque dominant leg and (H) peak torque nondominant leg measured at pre, 0.5 h post and 48 h post in Italian Serie A youth soccer players (*n* = 48). Individual responses are plotted with grey circles and lines, whereas mean responses are plotted with black dots and lines. Significant changes in comparison with baseline (pre) are indicated with asterisks (*p* < 0.05). CMJ: countermovement jump; h: hours; IPC: isometric posterior chain; RSI: reactive strength index.

CMJ measures were significantly reduced from pre to 0.5 h post‐match (mean change: from −2.7% to −6.5%; *d*: from 0.15 to 0.43 and *p* < 0.001) and returned to baseline levels at 48 h post‐match (*d*: from −0.09 to 0.05 and *p* > 0.05). IPC measures were significantly reduced from pre to 0.5 h post‐match (mean change: from −15.0% to −17.1%; *d*: from 0.83 to 1.19 and *p* < 0.001) but remained reduced at 48 h post‐match (mean change: from −7.2% to −7.8%; *d*: from 0.43 to 0.50 and *p* < 0.001). Perceived muscle soreness (*d*: 1.97 and *p* < 0.001) and fatigue (*d*: 1.72 and *p* < 0.001) were significantly increased from pre to 0.5 h post‐match. At 48 h post‐match, muscle soreness remained above baseline (*d*: 0.58 and *p* < 0.001), whereas fatigue returned to baseline levels (*d*: 0.11 and *p* = 1.000). Descriptive, inferential and effect size statistics of the recovery responses following match play are reported in Table 2. Individual responses of CMJ and IPC tests assessed at pre, 0.5 h post and 48 h post are reported in Figure 2.

**TABLE 2:** Recovery responses following match play in Italian Serie A youth soccer players (*n* = 48).

	Pre	0.5 h post	48 h post	Pre versus 0.5 h post			Pre versus 48 h post		
				Δ (95% CI)	*p*‐value	Cohen's *d*	Δ (95% CI)	*p*‐value	Cohen's *d*
Countermovement jump test									
Jump height (cm)	35.0 ± 5.3	33.4 ± 5.3*^S^	35.4 ± 5.5	−1.6 (−2.2; −0.9)	< 0.001	0.30	0.4 (−0.3; 1.0)	0.484	−0.07
Peak power (W)	3469 ± 592	3376 ± 599*^S^	3527 ± 624	−93 (−147; −40)	< 0.001	0.15	58 (−2; 111)	0.059	−0.09
Force at zero velocity (N)	1743 ± 246	1629 ± 255*^S^	1730 ± 289	−114 (−152; −77)	< 0.001	0.43	−13 (−51; 24)	1.000	0.05
RSI‐modified (m/s)	0.53 ± 0.09	0.50 ± 0.09*^S^	0.53 ± 0.11	−0.03 (−0.04; −0.01)	< 0.001	0.29	0.00 (−0.02; 0.02)	1.000	−0.01
Isometric posterior chain lower‐limb muscle test									
Peak force dominant leg (N)	300 ± 56	254 ± 51*^M^	276 ± 49*^S^	−46 (−55; −35)	< 0.001	0.87	−24 (−34; −13)	< 0.001	0.45
Peak force nondominant leg (N)	286 ± 43	237 ± 40*^M^	266 ± 40*^S^	−49 (−59; −39)	< 0.001	1.19	−20 (−31; −11)	< 0.001	0.50
Peak torque dominant leg (Nm)	116 ± 23	99 ± 21*^M^	107 ± 20*^S^	−17 (−22; −13)	< 0.001	0.83	−9 (−13; −5)	< 0.001	0.43
Peak torque nondominant leg (Nm)	111 ± 18	92 ± 17*^M^	103 ± 17*^S^	−19 (−23; −15)	< 0.001	1.09	−8 (−12; −4)	< 0.001	0.46
Perceptual scales									
Muscle soreness (cm)	1.9 ± 1.3	5.3 ± 1.9*^L^	2.9 ± 1.9*^S^	3.4 (2.7; 4.0)	< 0.001	1.97	1.0 (0.3; 1.7)	0.001	0.58
Fatigue (cm)	2.6 ± 1.3	5.0 ± 1.3*^L^	2.8 ± 1.5	2.3 (1.7; 3.0)	< 0.001	1.72	0.2 (−0.5; 0.8)	1.000	0.11

*Note:* Data are mean ± SD. S: small; M: moderate and L: large effect sizes compared with pre. Trivial effect sizes are not reported.

Abbreviations: Δ, mean change; AU, arbitrary units; CI, confidence interval; RSI, reactive strength index; SD, standard deviation.

*Significant change from baseline (pre and *p* < 0.05).

### 3.3 Effects of Different Training Load Interventions on Recovery Responses

**FIGURE 3:**
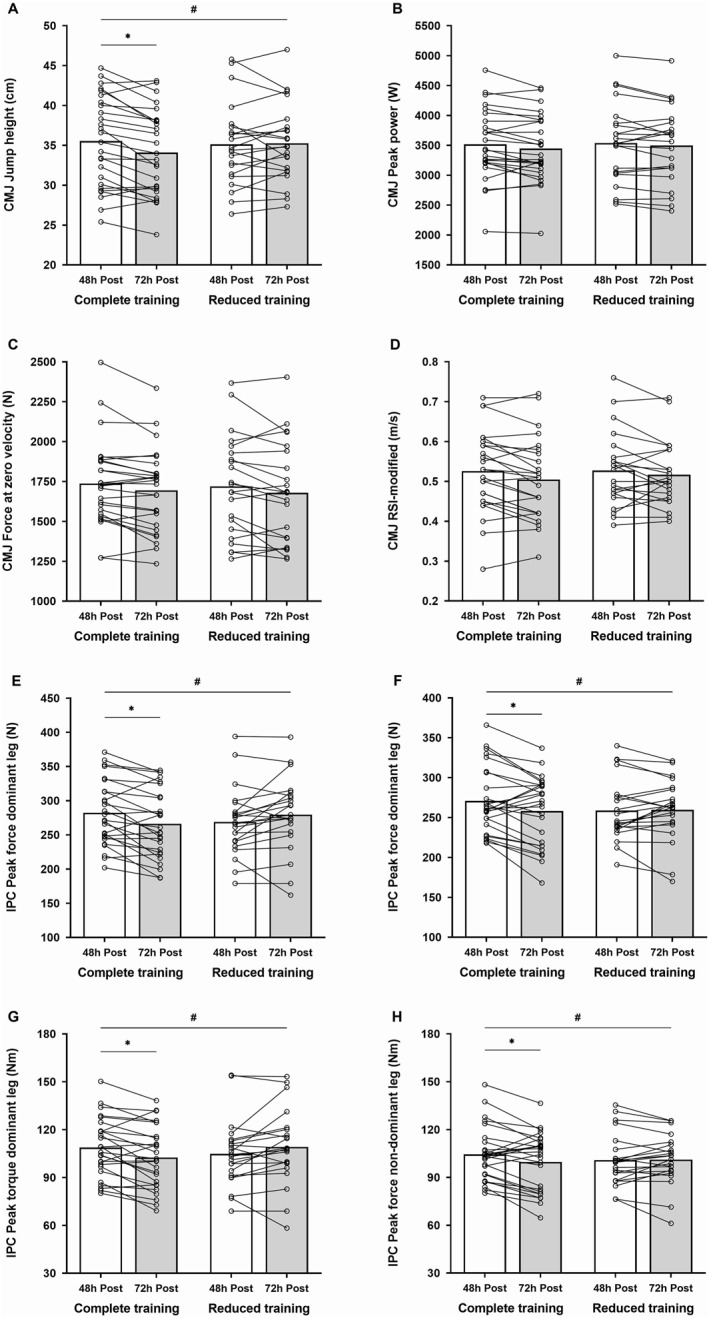
Individual (lines) and group (bars) responses to complete and reduce training load interventions in Italian Serie A youth soccer players on countermovement jump, (A) jump height, (B) peak power, (C) force at zero velocity, (D) RSI‐modified and isometric posterior chain, (E) peak force dominant leg, (F) peak force nondominant leg, (G) peak torque dominant leg and (H) peak torque nondominant leg measured at 48 h post (white bars) and 72 h post (grey bars). Significant changes in comparison with 48 h post are indicated with asteriks (*p* < 0.05), whereas significant time × group interactions are indicated with hash (*p* < 0.05). CMJ: countermovement jump; h: hours; IPC: isometric posterior chain; RSI: reactive strength index.

At 48 h post‐match, no significant differences were observed between groups for all recovery measures (*p* > 0.05). A significant time × group interaction was observed for CMJ height (*p* = 0.005), IPC peak force dominant leg (*p* < 0.001), IPC peak force nondominant leg (*p* = 0.033), IPC peak torque dominant leg (*p* < 0.001) and IPC peak torque nondominant leg (*p* = 0.037). From 48 h post to 72 h post, these physical performance measures were significantly reduced in the CT group (*p* < 0.05), whereas they remained unchanged in the RT group (*p* > 0.05). Conversely, following training load interventions, no significant time × group interaction was observed for CMJ peak power (*p* = 0.453), CMJ force at zero velocity (*p* = 0.331), CMJ RSI‐modified (*p* = 0.845), muscle soreness (*p* = 0.433) and fatigue (*p* = 0.088). The effects of complete and reduced training load interventions performed on MD + 2 on recovery responses are reported in Table 3. Individual responses to training load interventions are reported in Figure 3.

**TABLE 3:** Effects of complete and reduced training load interventions performed 48 h after match play on the recovery responses of Italian Serie A youth soccer players.

	Complete training (*n* = 26)		Reduced training (*n* = 22)		Main effect (*p*‐value)		
	Pre‐intervention (48 h post)	Post‐intervention (72 h post)	Pre‐intervention (48 h post)	Post‐intervention (72 h post)	Time × group	Time	Group
Countermovement jump test							
Jump height (cm)	35.5 ± 5.7	34.1 ± 5.3^a^	35.1 ± 5.2	35.3 ± 4.8	0.005^b^	0.016	0.806
Peak power (W)	3517 ± 591	3446 ± 563	3537 ± 675	3498 ± 646	0.453	0.011	0.843
Force at zero velocity (N)	1738 ± 272	1694 ± 258	1720 ± 314	1679 ± 317	0.918	0.002	0.845
RSI‐modified (m/s)	0.53 ± 0.10	0.51 ± 0.10	0.53 ± 0.10	0.52 ± 0.08	0.331	0.006	0.802
Isometric posterior chain lower‐limb muscle test							
Peak force dominant leg (N)	283 ± 48	266 ± 50^a^	269 ± 50	280 ± 54	< 0.001^b^	0.450	0.994
Peak force nondominant leg (N)	271 ± 42	258 ± 43^a^	259 ± 39	260 ± 38	0.033^b^	0.064	0.641
Peak torque dominant leg (Nm)	109 ± 19	103 ± 20^a^	105 ± 21	109 ± 23	< 0.001^b^	0.479	0.820
Peak torque nondominant leg (Nm)	104 ± 17	100 ± 18^a^	101 ± 16	101 ± 16	0.037^b^	0.067	0.829
Perceptual scales							
Muscle soreness (cm)	2.8 ± 2.2	3.0 ± 2.3	2.9 ± 1.6	2.7 ± 2.1	0.433	0.984	0.790
Fatigue (cm)	2.5 ± 1.5	2.8 ± 1.4	3.0 ± 1.4	2.9 ± 1.6	0.088	0.571	0.464

*Note:* Data are mean ± SD.

Abbreviations: AU, arbitrary units; CI, confidence interval; MD, match day; RSI, reactive strength index; SD, standard deviation.

^a^ Significant within‐group change from pre‐intervention (48 h post and *p* < 0.05).

^b^ Significant time × group interaction (*p* < 0.05).

## 4 Discussion

The aim of this study was to describe the recovery responses following match play and to examine the effects of two different training load interventions administered 48 h post‐match on recovery in Italian Serie A youth soccer players. Based on current evidence, this may be the first study to concurrently examine post‐match recovery responses and the impact of manipulating training load 2 days after match play in high‐level youth soccer players. The primary results demonstrated that an 80 min youth soccer match significantly impaired physical performance and perceptual recovery both immediately and in the subsequent days. Recovery patterns showed variation, with CMJ performance and perceived fatigue returning to baseline levels 48 h post‐match, whereas hamstring muscle force and perceived muscle soreness had not fully recovered by that time. When resuming training 48 h post‐match, a session characterised by high volume and high intensity adversely affected physical performance 72 h post‐match compared to a moderate‐intensity session. Nevertheless, the training intervention did not alter the perceived recovery of these high‐level youth players.

An 80 min soccer match significantly reduced CMJ performance, hamstring strength and increased muscle soreness and fatigue, showing acute fatigue in youth players. CMJ measures showed significant reductions ranging from −3% to −8% at post‐match. The magnitude of change observed in CMJ height (−5%; small effect) is consistent with previous studies (Barreira et al. 2023; de Hoyo et al. 2016; Izquierdo et al. 2020; Martin‐Garetxana et al. 2024; Romagnoli et al. 2016). However, the observed reduction in CMJ peak power (−3%; trivial effect) was larger than that reported in similar research on youth soccer players (Romagnoli et al. 2016). Although frequently documented, CMJ height and peak power may display post‐match and post‐training changes within their typical variation (Franceschi et al. 2023; Malone et al. 2015; Thorpe et al. 2017). Conversely, CMJ measures derived from the analysis of force‐time curve, such as force at zero velocity and RSI‐modified in the current study (−7% and −6%, respectively; small effect), demonstrated a higher probability of change following soccer match play. Previous research has shown marked variations in the eccentric force components of the CMJ in responses to youth soccer match play (de Hoyo et al. 2016; Springham et al. 2024). These observations suggest that CMJ eccentric force components may better reflect the neuromuscular reduction and the movement strategy adjustments following soccer competitions.

Hamstring muscle function was also significantly impaired at post‐match, with reductions in IPC peak force and peak torque for both dominant and nondominant legs ranging from −15% to −17% (moderate effect). The magnitude of post‐match changes aligns with previous studies assessing acute changes following 90 min matches in youth soccer players (Constantine et al. 2019; Wollin et al. 2017), indicating the high involvement of hamstring muscles in youth soccer match play. Perceived muscle soreness and fatigue exhibited similar acute responses compared to the pre‐match condition. This also agrees with the impaired perceptions of recovery and fatigue observed in youth soccer players after competitive matches (De Ste Croix et al. 2019; Martin‐Garetxana et al. 2024; Romagnoli et al. 2016). These combined acute changes in physical performance and perceptions of fatigue likely reflect perturbations in the central nervous system and peripheral muscle function, the depletion of muscle glycogen levels and the onset of match‐induced muscle damage, commonly observed at the end of soccer matches and intense training sessions (Brownstein et al. 2017; Deely et al. 2022; Mohr et al. 2022). In particular, the reduction of the force‐generating capacity of the hamstring muscles accompanied by increased perceived muscle soreness can be attributed to the muscle damage experienced by high‐level youth soccer players following soccer match play (Pooley et al. 2020).

Following acute impairments of physical performance and perceptual state after match play, recovery responses exhibited distinct patterns. Although CMJ and perceived fatigue recovered 48 h post‐match, IPC and perceived muscle soreness were still not fully recovered. Our data indicated a complete recovery of CMJ performance 48 h post‐match in all measures analysed. Previous studies on youth soccer have yielded mixed outcomes regarding CMJ performance; under‐19 Spanish and Italian players exhibited a sustained decrease in jump height (−3% to −6% from baseline) 48 h post‐match (de Hoyo et al. 2016; Romagnoli et al. 2016). In contrast, similar to our findings, under‐19 Portuguese and under‐18 English players showed complete CMJ recovery at this timeframe (Barreira et al. 2023; Springham et al. 2024). This recovery kinetics also aligns with measures of central and peripheral fatigue, which showed a return to baseline values 48 h after the match in adult soccer players (Brownstein et al. 2017). Collectively, these findings suggest that the time to restoration of CMJ appears to be shorter in youth soccer players than those observed in the adult population (Silva et al. 2018).

Conversely, hamstring muscle function remained impaired 48 h post‐match. IPC peak force and torque did not return to baseline values in either the dominant and nondominant legs (−8% and −7%, respectively), with significant changes of small effect compared to pre‐match. Although some studies reported a return to baseline in high‐level youth soccer players within this timeframe (Constantine et al. 2019; Springham et al. 2024; Wollin et al. 2017), more recent findings by Barreira et al. (2023) align with our results, documenting impaired posterior‐chain muscle function 2 days after a match in under‐19 Portuguese players. Our data suggest that youth players require a longer recovery period for hamstring muscle function, similar to professional adult players who show prolonged recovery times up to 72 h post‐match (Silva et al. 2018). The current study found that moderate reductions in hamstring function persist 48 h post‐match in youth players, likely due to the heavy involvement of hamstring muscles during high‐intensity actions and eccentric activities which induces muscle damage in the days post‐match (Carmona et al. 2024). Forty‐eight hours after the match, hamstring muscle force had not fully recovered and perceived muscle soreness remained high, indicating incomplete recovery. Previous studies have recorded increased muscle damage markers 48 h post‐match in adolescent players (de Hoyo et al. 2016; Romagnoli et al. 2016), indicating that the recovery time reflects both mechanical stress from eccentric contractions and metabolic fatigue caused by match play (Thorpe 2021). These findings indicate that youth soccer players' performance capacity may not be fully restored 2 days after an 80 min match play due to the altered perception of muscle soreness and the reduced hamstring muscle function.

The complete training session characterised by high‐volume and high‐intensity load adversely affected physical performance responses compared to the reduced moderate‐intensity training session. Our findings reveal substantial differences among training load interventions (moderate to very large effect in the training load sustained on MD + 2), leading to significant small reductions in CMJ height and all IPC measures from 48 to 72 h post‐match in the complete training group. These outcomes are consistent with previous research reporting better restoration of knee flexor muscle force production and lower creatine kinase levels following an active recovery session 48 h post‐match compared to a typical training session (Trecroci et al. 2020, 2021). Similarly, an active recovery protocol performed immediately after the match showed improved recovery responses 48 h after the match in under‐18 English soccer players compared to a static stretching protocol (Pooley et al. 2020). Despite the impact on physical performance capacity, our training load interventions did not influence the perceived recovery state and the other CMJ measures. This result seems to reflect the lower physical and physiological demands experienced by the players during training sessions compared to match demands. Conversely, the reduced training group did not show declines in physical performance and perceived recovery from 48 to 72 h post‐match, suggesting that a moderate‐training session (sRPE: 3.3 ± 0.8 AU) allows the maintenance of performance capacity early in the microcycle.

Despite practitioners' inclination towards prioritising recovery within the initial 48 h post‐match window in elite adult soccer (Buchheit et al. 2021; Cross et al. 2019), published research has shown that youth soccer players can be subjected to elevated training loads 2 days post‐match (Franceschi et al. 2024; Wrigley et al. 2012). In this view, the current study provides new insights into the effects of soccer‐specific training on recovery in high‐level youth soccer players. Taken together, the results of this research suggest that on MD + 2 adolescent players' performance capacity may not be fully restored, and that a high‐volume and high‐intensity training session administered within this time point might further affect the recovery of performance 72 h post‐match. However, given the small effect sizes observed, it is difficult to assert whether the additional training load imposed on the CT group impeded the recovery process to a critical degree. Manipulation of training load can serve as a practical tool to affect acute performance and recovery responses across the microcycle in line with previous research conducted with team sport athletes (Douchet et al. 2022; Douchet et al. 2024; Slattery et al. 2012). Therefore, adjusting the training load in the days after match play may influence recovery of performance capacity and subsequent readiness to train of youth soccer players.

Although our study provides insights into post‐match recovery responses of high‐level youth soccer players, it has limitations that should be acknowledged. Firstly, we did not assess the long‐term effects of the training load manipulation on subsequent match performance and training effects over the microcycle (Slattery et al. 2012). Given that balancing training stimulus and recovery is a fundamental aspect of the training process, exposure to appropriate training under conditions of incomplete recovery might also enhance robustness and subsequently promote chronic adaptations in youth soccer players. Secondly, although the decision to use a friendly match play enhanced ecological validity and increased sample size, it also resulted in less control over the match stimulus compared to a match‐simulation protocol (Field et al. 2022). Additionally, incorporating physiological markers, such as muscle damage and inflammation, could provide a more comprehensive understanding of the recovery process of youth soccer players. Future research should examine the effects of recovery‐based and training‐based strategies adopted in the 2 days following the match using cross‐over and repeated measures designs (Hecksteden et al. 2022).

## 5 Conclusion

An 80 min soccer match significantly impaired both physical performance and perceptual recovery in Italian Serie A youth players. Recovery responses exhibited varied timelines, with CMJ and perceptual fatigue returning to baseline levels within 48 h post‐match, whereas hamstring muscle force and perceptions of muscle soreness were not fully recovered. Additionally, a high‐volume and high‐intensity training session conducted 2 days after the match may further impair the recovery of physical performance compared to a moderate training session. These findings underscore the necessity of carefully managing training loads in the days following a match to ensure recovery of performance capacity.

## Conflicts of Interest

The authors declare no conflicts of interest.

## Supporting information

Table S1
