# Supplementary material for: Post‐Match Recovery Responses in Italian Serie A Youth Soccer Players: Effects of Manipulating Training Load 48 h After Match Play
Source: Eur J Sport Sci. 2025 Apr 20;25(5):e12297. doi: 10.1002/ejsc.12297 (PMC12010046; doi:10.1002/ejsc.12297)
Supplement: Supplementary file 1 — Table S1 [file EJSC-25-e12297-s001.docx]

**Supplementary material**. Match load characteristics: descriptive, inferential and effect size statistics of external and internal load measures between complete and reduced training groups. Data are mean ± SD.

|  | **Complete training (n = 26)** | **Reduced training (n = 22)** | **Mean difference (95% CI)** | **p-value** | **Cohen's d (95% CI)** | **Interpretation** |
| --- | --- | --- | --- | --- | --- | --- |
| **External load** |  |  |  |  |  |  |
| Total distance (m) | 11248 ± 719 | 11041 ± 787 | 207 (-230; 645) | 0.345 | 0.28 (-0.3; 0.85) | *small* |
| Distance > 15 km/h (m) | 1823 ± 375 | 1746 ± 374 | 77 (-141; 296) | 0.478 | 0.21 (-0.36; 0.78) | *small* |
| Distance > 20 km/h (m) | 560 ± 200 | 525 ± 211 | 35 (-84; 155) | 0.554 | 0.17 (-0.4; 0.74) | *trivial* |
| Distance > 25 km/h (m) | 114 ± 74 | 104 ± 94 | 10 (-39; 59) | 0.692 | 0.12 (-0.45; 0.68) | *trivial* |
| Maximal speed (km/h) | 29.8 ± 1.9 | 29.6 ± 2.5 | 0.2 (-1.1; 1.5) | 0.757 | 0.09 (-0.48; 0.66) | *trivial* |
| Accelerations > 3 m/s^2^ (count) | 71 ± 19 | 68 ± 14 | 3 (-8; 12) | 0.661 | 0.13 (-0.44; 0.70) | *trivial* |
| Decelerations < -3 m/s^2^ (count) | 80 ± 19 | 73 ± 20 | 7 (-4; 19) | 0.216 | 0.36 (-0.21; 0.93) | *small* |
| **Internal load** |  |  |  |  |  |  |
| Average HR (bpm) | 158 ± 12 | 148 ± 20 | 10 (-0.0; 20) | 0.056 | 0.65 (-0.01; 1.30) | *moderate* |
| Peak HR (bpm) | 196 ± 8 | 190 ± 12 | 6 (-1; 12) | 0.079 | 0.57 (-0.07; 1.19) | *small* |
| Time > 85% HR_max_ (min) | 42 ± 18 | 32 ± 22 | 10 (-3; 22) | 0.117 | 0.50 (-0.12; 1.11) | *small* |
| Time > 90% HR_max_ (min) | 18 ± 14 | 15 ± 16 | 3 (-6; 12) | 0.502 | 0.21 (-0.40; 0.82) | *small* |
| sRPE (AU) | 5.6 ± 1.0 | 5.5 ± 1.0 | 0.1 (-0.4; 0.7) | 0.624 | 0.14 (-0.43; 0.71) | *trivial* |
| sRPE-TL (AU) | 587 ± 101 | 571 ± 91 | 16 (-40; 72) | 0.572 | 0.17 (-0.41; 0.73) | *trivial* |
| Abbreviations: CI: confidence interval; m: meters; n: number; HR: heart rate; bpm: beats per minute; TL: training load; AU: arbitrary units. | | | | | | |
